# Higher freshwater fish and sea fish intake is inversely associated with colorectal cancer risk among Chinese population: a case-control study

**DOI:** 10.1038/srep12976

**Published:** 2015-08-12

**Authors:** Ming Xu, Yu-Jing Fang, Yu-Ming Chen, Min-Shan Lu, Zhi-Zhong Pan, Bo Yan, Xiao Zhong, Cai-Xia Zhang

**Affiliations:** 1 Department of Medical Statistics and Epidemiology, School of Public Health, Sun Yat-sen University, Guangzhou 510080, China; 2 Department of Colorectal Surgery, Sun Yat-sen University Cancer Center, State Key Laboratory of Oncology in South China, Collaborative Innovation Center for Cancer Medicine, 651 Dongfeng Road East, Guangzhou 510060, China; 3 Department of Experimental Research, Sun Yat-sen University Cancer Center, State Key Laboratory of Oncology in South China, Collaborative Innovation Center for Cancer Medicine, 651 Dongfeng Road East, Guangzhou 510060, China

## Abstract

The association between specific fish intake and colorectal cancer risk remains controversial. This study aimed to examine the association between specific fish intake and colorectal cancer risk in Chinese population in a large case control study. During July 2010 to November 2014, 1189 eligible colorectal cancer cases and 1189 frequency-matched controls (age and sex) completed in-person interviews. A validated food frequency questionnaire was used to estimate dietary intake. Multivariate logistical regression models were used to estimate the odds ratio (OR) and 95% confidence interval (95% CI) after adjusting for various confounders. A strong inverse association was found between freshwater fish intake and colorectal cancer risk. Compared with the lowest quartile, the highest quartile intake showed a risk reduction of 53% (OR 0.47, 95% CI = 0.36–0.60, *P*_*trend*_ < 0.01) after adjustment for various confounders. The inverse association were also observed for sea fish (OR 0.79, 95%CI = 0.62–0.99, *P*_*trend*_ < 0.01) and fresh fish (OR 0.49, 95%CI = 0.38–0.62, *P*_*trend*_ < 0.01). No statistically significant association was found between dried/salted fish and shellfish intake and colorectal cancer risk. These results indicate that higher consumption of freshwater fish, sea fish and fresh fish is associated with a lower risk of colorectal caner.

Fish is part of the usual diet of most people worldwide. It has a richer content of n-3 fatty acids which have been reported to suppress mutations, inhibit cell growth and enhance cell apoptosis, thus inhibiting colon carcinogenesis^1^,^2^. Fish is also a source of vitamin D, which has been reported to be inversely associated with colorectal cancer^3^. Selenium, another nutrient contained in fish, has been shown to exert anticancer effects in *in vitro*, animal and human studies^4^,^5^.

Though laboratory and animal studies suggest that consumption of fish inhibits carcinogenesis, the results of epidemiologic data have been inconclusive. A systematic review of five cohort studies and 12 case-control studies among Japanese population showed insufficient evidence of a protective effect of fish consumption against colorectal cancer risk^6^. However, a meta-analysis published in 2012 with 22 prospective cohort and 19 case-control studies found that fish consumption decreased the risk of colorectal cancer^7^.

Though many studies have evaluated the effect of fish intake on colorectal cancer, most have been conducted in the United States or Europe. In China, only the Shanghai Women’s Health Study (SWHS) has examined the association between fish intake and the risk of colorectal cancer and found no association between total fish intake and colorectal cancer risk^8^,^9^. Fish is a major source of animal food in the traditional diet among the coastal regions of mainland China, with a mean intake of 90–200 g/day (raw weight) in Chinese coastal areas according to the 2002 National Nutritional Survey^10^. Moreover, consumption of fish in Guangdong is higher and steaming is the most frequently used cooking method^11^. Furthermore, previous studies abroad just focus on the association between total fish intake and colorectal cancer, but not to carry out in-depth study of the different types of fish. Different types of fish contain different nutrients^12^, which may play different roles in the relationship with colorectal cancer. Thus, we performed a case control study on the association between different types of fish and colorectal cancer risk in Guangdong, a coastal province in the southern part of China. The valid dietary assessment as well as the large sample size provided us an opportunity to examine the association between fish and risk of colorectal cancer according to sex, anatomic site of tumor and sort of fish.

## Results

Table 1 presents the socio-demographic characteristics of study subjects and the distribution of selected colorectal caner risk factors. Characteristics of hospital-derived controls and community-derived controls are also shown in Table 1. Of the 1189 cases, 663 were males while 526 were females. Seven hundred and twenty-one (419 males and 302 females) were classified to have colon cancer and 468 (244 males and 224 females) were classified to have rectal cancer. Compared to the controls, case subjects had greater household income, lower education level, less physical activity and were more likely to have a family history of cancer and regular smoking and passive smoking experience. In addition, the occupational status and marital status differed between cases and controls. All of the above variables were considered potential confounders and adjusted for in subsequent analysis. No significant differences were found between cases and controls in alcohol drinking, BMI, and menopausal status (in female subgroup).

The comparison results of consumption of specific fish and total energy intake between cases and controls are displayed in Table 2. Compared to control subjects, cases had lower intakes of freshwater fish, sea fish and fresh fish. No significant difference was found in dried/salted fish and shellfish intake between the cases and controls.

As shown in Table 3, freshwater fish intake was found to be inversely associated with colorectal cancer risk. Compared with the lowest quartile, the highest quartile of freshwater fish intake showed a risk reduction of 53% (OR = 0.47, 95%CI = 0.36–0.60, *P*_trend_ < 0.01) after adjustment for potential dietary and non-dietary confounding variables. The inverse association was also observed for sea fish and fresh fish, with adjusted ORs (95% CI) of 0.79 (0.62–0.99) for sea fish and 0.49 (0.38–0.62) for fresh fish comparing the highest quartile with the lowest quartile. No statistically significant association was observed between dried/salted fish and shellfish intake and colorectal cancer risk.

A sex-stratified analysis showed that the inverse association between freshwater fish, fresh fish and colorectal cancer risk were observed in both males and females (Table 4). However, the inverse associations between sea fish intake and colorectal cancer risk were only observed in females. The adjusted ORs (95% CIs) were 0.59 (0.41–0.84) in females and 1.02 (0.73–1.41) in males compared the highest with the lowest sea fish quartiles. Interaction analysis between sex and colorectal cancer risk showed that the association differed significantly in all other specific fish otherwise freshwater fish and dried/salted fish intake stratified by sex.

Subgroup analysis by cancer site showed that the inverse associations between freshwater fish, fresh fish and colorectal cancer risk were observed in both sites. However, the inverse association between sea fish intake and colorectal cancer risk was only observed in rectal cancer, but not in colon cancer, with the adjusted ORs (95% CIs) of 0.66 (0.48–0.91) in rectal cancer and 0.91 (0.70–1.20) in colon cancer compared the highest with the lowest quartile (Table 5). Subgroups analysis by source of controls did not differ between total fresh fish intake and colorectal cancer risk (data not shown). The associations of fish intake with colorectal cancer risk did not differ significantly stratified by socioeconomic status (income level and education level)(data not shown)

## Discussion

This large case control study, with 1189 cases and 1189 controls, examined the association between fish intake and colorectal cancer risk among Guangdong Chinese population. The results showed that higher intake of fresh fish including freshwater fish and sea fish was associated with a lower risk of colorectal cancer.

Some previous studies have supported the hypothesis that a greater intake of fresh fish is favorable for the prevention of colorectal cancer^7^,^13^,^14^,^15^,^16^,^17^,^18^, which is consistent with the results of the present study. A meta-analysis published in 2012 with 22 prospective cohort and 19 case-control studies found that fish consumption decreased the risk of colorectal cancer by 12% (summary OR = 0.88, 95%CI = 0.80–0.95)^7^. Another meta-analysis published in 2007 based on 14 prospective cohort studies also reported that the pooled relative risk for the highest compared with the lowest fish consumption category were 0.88 (95% CI = 0.78–1.00) for colorectal cancer incidence^18^. Results from the Physicians’ Health Study (PHS) followed for 22 years suggest that intake of fish may decrease the risk of colorectal cancer^16^. Reports from the European Prospective Investigation into Cancer and Nutrition (EPIC)^14^, the Cancer Prevention Study II (CPS II)^13^ and a hospital-based case-control study in Eastern Europe^19^ also indicated an inverse association.

Whereas some other studies did not support the notion that high fish intake might decrease the risk of colorectal cancer^6^,^20^,^21^,^22^,^23^,^24^. The results of the SWHS indicated that fish intake was not related to the risk of colorectal cancer, which may be attributed to the effect of water pollution^8^,^9^. A high concentration of DDT in spiny-head croaker, trident goby, and pike eel collected from Hangzhou Bay, south of Shanghai was reported^25^. Fish, particularly shellfish raised in industrial areas such as Shanghai, may have a high level of methyl mercury, polychlorinated dibenzo-p-dioxins and dibenzofurans, organochlorine residues and other chemicals, some of which have been shown to be mutagens or animal carcinogens^26^. Another possible explanation for this difference is that the frequency, amount and sort of fish consumed differed by different regions. People in Guangdong province may consume more fish than people in Shanghai. In the present study, the mean fresh fish intake in control subjects is 77.51 g/day, which is about 1.5 times greater than that of fish consumption in Shanghai (50.6 g/day)^8^. The inconsistent results across studies could also be explained by the type of fish consumed. Some studies did not distinguish fresh fish from dried/salted fish which may be positively association with the risk of colorectal cancer^27^,^28^.

Evidence from animal and *in vitro* studies indicates that n-3 fatty acids present in fatty fish and fish oils may inhibit carcinogenesis. Fish oil or n-3 fatty acids inhibit chemically induced colorectal carcinogenesis in rodents^29^,^30^. High intake of n-3 fatty acids suppresses the production of arachidonic acid-derived eicosanoids such as prostaglandin E_2_ and leukotriene B_4_^31^. N-3 fatty acids could also suppress the expression of inducible nitric oxide synthetase (NOS) and nuclear transcription factor κB (NF-κB)^31^. Selenium, another important nutrient contained in fish, has been shown to reduce the incidence of cancer through several anticarcinogenic pathways, including prevention and repair of oxidative DNA damage, alteration of metabolism of carcinogenic agents and regulation of immune response^32^,^33^,^34^,^35^. It is also possible that selenium could cause adenomas to regress, shrink, or grow more slowly instead of preventing their initial growth^4^. Fish is a rich source of Vitamin D. Vitamin D directly alters patterns of gene expression via the vitamin D receptor and influences the outcome between proliferation, differentiation or apoptosis, which may help preventing initiation as well as progression of colorectal cancer^36^.

In the current study, the inverse association between freshwater fish intake and colorectal cancer risk was found to be stronger than that of the sea fish and colorectal cancer. The mean long chain n-3 fatty acid intake from freshwater fish and sea fish in control subjects was 23.42 mg/day and 26.92 mg/day, respectively. Although sea fish contains higher amounts of long chain n-3 fatty acids than dose freshwater fish; this did not seem to support the hypothesis that long chain n-3 fatty acids are responsible for the protective effect of sea fish on colorectal cancer risk. Moreover, more than 75 percent cases took sea fish below the average level, which might limit the power to detect a stronger association in relation to sea fish intake. The mean intake of α-linolenic acid from freshwater fish and sea fish in the present study was 28.77 mg/day and 13.70 mg/day, respectively. And the proportion of freshwater fish-derived n-3 fatty acid to total fat was significantly higher than the proportion of n-3 fatty acid from sea fish to total fat (0.10% *versus* 0.03%). We have previously reported that consumption of n-3 fatty acid and α-linolenic acid was inversely associated with the risk of colorectal cancer^37^. Therefore, relatively higher daily intake of α-linolenic acid from freshwater fish might contribute to a stronger inverse association with colorectal cancer risk, though the exact mechanism underlying the protective effect of α-linolenic acid is unclear. In addition, freshwater fish provides a healthy source of high-quality proteins, essential vitamins and minerals. In a test with spontaneously hypertensive rats, which received a diet containing 10% freshwater fish oil, development of hypertonia was clearly delayed^38^. Therefore, though less research has been devoted to the significance of relationship between freshwater fish and its value for human nutrition, some animal experiments suggest that the nutritive quality of freshwater fish is even better and it may benefit the prevention of cancers in human.

A high consumption of dried or salted fish has been linked to an increased risk of colorectal cancer in some studies^27^,^28^. Possible reason is that dried/salted fish, as a kind of preserved foods, contains *N*-nitrosodimethylamine and other volatile *N*-nitroso compounds^39^ that show mutagenicity^40^,^41^ and carcinogenicity in laboratory animals^42^. However, the present study found no significant association between dried/salted fish and colorectal cancer risk. It may be that consumption of preserved foods has declined after the popularization of refrigerators in China since the 1980s^10^, therefore, the impact of dried/salted fish intake on the risk of colorectal cancer appears small.

Stratified analysis by sex showed that the association of sea fish intake with colorectal cancer risk seemed to be more pronounced in females than in males. Sex differences in associations between fish intake and colorectal cancer risk have been noted in some previous studies^18^,^20^,^43^. In agreement with our result, a meta-analysis^18^ showed that the pooled relative risk for colorectal cancer incidence was more pronounced for females and in studies with a large exposure contrast. One possible explanation may be that the amount of sea fish intake was higher in females than in males. Moreover, endogenous estrogen may alter the normal n-3 fatty acids metabolism through changes in fatty acids utilization and oxidation^20^. However, a case reference study in Japan^43^, found that the protective effect was observed for frequent intake of fish and risk of colorectal cancer in males but not in females. More studies are needed to clarify this issue.

The protective effect of sea fish consumption is more prominent in rectal cancer than that in colon cancer, which might be accounted for by the different characteristics between colon and rectal cancer. This finding is consistent with a previous meta-analysis, in which the summary OR of the highest *versus* the lowest quartile of fish intake was 0.79 (95% CI = 0.65–0.97) for rectal cancer, while the OR was 0.96 (95% CI = 0.81–1.14) for colon cancer^7^. Colon cancers are generally molecularly heterogeneous, whereas rectal cancers mostly arise via a single neoplastic pathway^44^,^45^. And it is well known that the therapeutic strategies for colon cancer and rectal cancer differ^46^. Despite this, further exploration is needed to elucidate the exact mechanism at play.

There is relatively lager sample size and we have enough power to detect small associations in the risk of colorectal cancer. However, we should also admit that there are some limitations of the present study. First, selection bias is a potential limitation in case-control studies. Colorectal cancer patients were recruited from only one hospital, Sun Yat-sen University Cancer Center. However, this is the largest cancer center in the South China. Reports also indicated that the colorectal cancer patients in this hospital had the same clinical features as patients in other two big hospitals^47^ in Guangdong province and those in mainland China^48^. Moreover, the similar results of the hospital-based controls and population-based controls suggested that the selection of controls did not have an influence on the results. Furthermore, although cases had relatively higher household income and lower education level than controls, stratified analysis by socioeconomic status showed no interaction between socioeconomic status and fish intake. And socioeconomic status is unlikely to have a strong influence on the fish on colorectal cancer association. Therefore, the possibility of selection bias should be reduced in our study. Second, fish intake was assessed only once on the past 12-month questionnaire, and this measure may not be representative of fish intake over time. However, the non-differential misclassification errors observed in the FFQ likely attenuate the estimated OR toward the null and true ORs are probably greater than they seem. Third, as in any case-control studies, the potential for recall bias exists in our study. To minimize this bias, we tried to interview cases as soon as the diagnosis was made. The average interval between diagnosis and interview for cases was 11.4 days. We also provided photographs of foods with usual portion size to help participants accurately estimate the food intake. Fourth, there were also potential confounders that were unable to be considered, and therefore, residual confounding might also remain even though various dietary and non-dietary confounders were adjusted.

In conclusion, this large case control study suggest that higher intake of fresh fish including freshwater fish and sea fish is inversely associated with the risk of colorectal cancer.

## Methods and Materials

### Study subjects

This is an ongoing case-control study beginning in July 2010 in Guangdong province of China for the purpose of examining the relationship between lifestyle factors and colorectal cancer risk. The selection of cases and controls has been described in detail elsewhere^37^,^49^,^50^. Briefly, during July 2010 and November 2014, a total of 1192 cases out of 1322 eligible histologically confirmed incident colorectal cancer patients were successfully interviewed from Sun Yat-sen University Cancer Center, Guangzhou, China, with a participation rate of 90.2%. Except that the total energy intake was quite low in three cases (411 kcal/day, 530 kcal/day, 549 kcal/day), we finally recruited 1189 cases in our analysis.

Control subjects were recruited from: (1) inpatients of three affiliated hospitals of Sun Yat-sen University during the same period as the cases subjects. At last, 611 control subjects were selected from the Department of Ophthalmology, Ear-Nose-Throat, Plastic and Reconstructive Surgery, and Vascular Surgery with the disease of eye disorders, ear-nose-throat disease, trifacial neuralgia, varicose veins, osteoarthritis, degenerate joint disease, orthopedics, facial paralysis, and acute appendicitis. Totally, 699 hospital-derived controls were identified and 611 were successfully interviewed, with a participation rate of 87.4%; and (2) the other 578 control subjects were obtained from the apparently healthy community residents in the same cities invited through a variety of strategies such as written invitations, flyers, or referrals. Eligibility criteria for controls were the same as described for the cases except that they had no history of any cancers. They were frequency matched by sex, age (5-year interval) and residence (rural/urban) to the case subjets.

The present study was approved by the ethical committee of School of Public Health of Sun Yat-sen University. Signed informed consent forms were obtained from all participants prior to the interview and the methods were carried out in accordance with the approved guidelines.

### Data collection

All study participants completed a face-to-face interview by trained interviewers using a structured questionnaire to collect information on dietary habits and potential confounding factors. The core questionnaire asked for data on social-demographic characteristics, body weight and height, active and passive smoking, alcohol intake, physical activity, family history of cancer in first-degree relatives and prior disease history for all subjects. Menstrual and reproductive factors were also obtained for female subjects. Relevant medical information, medical diagnosis, and histological findings were abstracted from medical records. Body mass index (BMI) was calculated by dividing weight (kg) by height squared (m^2^). In the current study, regular smoker was defined as someone smoking at least 1 cigarette a day for no less than 6 consecutive months. Passive smoking was designated as exposure to others’ tobacco smoke for at least 5 min per day in previous 5 years. Regular drinking was defined as alcohol drinking at least once per week during the past year. Postmenopausal status was defined as at least 12 months since the last menstrual cycle. In addition, physical activity was specified as leisure-time physical activity.

Food consumption data were collected by an 81-item food-frequency questionnaire (FFQ). Participants were asked to report information on frequency of intake and portion size during the preceding 12 months prior to diagnosis for cases or interview for controls, which was used to calculate the average intake of each food item in grams per day. A commonly used portion size was specified for each food (e.g., bowl, slice, glass, or unit, such as one apple or one banana). For vegetables and animal foods, a liang (1 liang = 50 g), a common weight measure familiar to the study subjects, was used to estimate the usual portion size. Food photographs with usual intake portion size were also provided to help participants estimate and record the amounts of food consumed. Total energy and other nutrients intakes were computed based on the 2002 Chinese Food Composition Table^51^.

In this analysis, the intake of fish was assessed using five items including freshwater fish (e.g., grass carp, black carp, bullhead, crucian carp, mandarin fish, etc), sea fish (e.g., pomfret, grouper, golden threat fish, hairtail, etc.), small fish or canned fish eaten with bones, salted fish, shrimp and crab, and other crustaceans and mollusks. Based on the similarities in nutrient composition, fish were classified into five groups: freshwater fish, sea fish, dried/salted fish, shellfish and fresh fish. In detail, dried/salted fish included small fish or canned fish eaten with bones and salted fish. Shellfish consisted of shrimp and crab, other crustaceans and mollusks. Fresh fish was considered to be the sum of freshwater fish, sea fish and shellfish. Development and validation of the FFQ was described elsewhere and this FFQ has been used in previous studies^52^.

### Statistical analysis

Statistical analysis was carried out using SPSS software (version 21.0). Fish consumptions were adjusted for total energy intake by the regression residual method^53^. Subjects were divided into quartiles (Q1–Q4) based on the distribution of fish intake among the control group. Continuous variables (such as age, dietary fish intake) were compared between cases and controls using a Student’s t test while categorical variables (such as income, educational level, smoking status) were analyzed using a chi-square test. Unconditional logistic regression models were used to estimate the odds ratios (ORs) and 95% confidence intervals (CIs) for the associations between fish intake and the risk of colorectal cancer, with the lowest quartile as the reference group. The ORs and 95% CIs were further adjusted for potential confounders by the following variables: age, sex, education, marital status, occupation, income, family history of cancer, smoking status, passive smoking, alcohol drinking, physical activity, BMI, and menopausal status (in female subjects). Tests for trend were performed by entering the categorical variables as continuous variables in the multiple regression models.

Stratified analysis was conducted to assess the associations of fish intakes with colorectal cancer risk modified by sex. The interaction between sex and fish intake in relation to colorectal cancer risk was evaluated by multivariate logistic regression. Subgroups analysis by cancer site (colon or rectal cancer) and source of controls (hospital or community) were also conducted. Since socioeconomic factors were not well-balanced between cases and controls in the present study, stratified analysis by socioeconomic status (education level and income level) were also conducted. All *P* values were 2-sided and statistical significance was determined at the *P* < 0.05 level in the present study.

## Additional Information

**How to cite this article**: Xu, M. *et al.* Higher freshwater fish and sea fish intake is inversely associated with colorectal cancer risk among Chinese population: a case-control study. *Sci. Rep.* **5**, 12976; doi: 10.1038/srep12976 (2015).

## Figures and Tables

**Table 1:** Demographic and selected risk factors of colorectal cancer cases and controls in Chinese population.

	Cases(n = 1189)	Controls(n = 1189)	*P-*valuebetweenCases andcontrols	Community-derivedcontrols(*n* = 611)	Hospital-derived controls(*n* = 578)	*P-*value between control groups
Age, yr, (mean ± SD)	56.8 ± 14.0	56.3 ± 10.0	0.31	62.0 ± 6.2	50.4 ± 9.7	<0.01
Marital status (n, %)			0.02			0.02
Married	1131 (95.1)	1103 (92.8)		556 (91.0)	547 (94.6)	
Unmarried/divorces/widowed	58 (4.9)	86 (7.2)		55 (9.0)	31 (5.4)	
Residence (n, %)			1			0.97
Urban	781 (65.7)	781 (65.7)		401 (65.6)	380 (65.7)	
Rural	408 (34.3)	408 (34.3)		210 (34.3)	198 (34.3)	
Educational Level (n, %)			<0.01			<0.01
Primary school or below	376 (31.6)	242 (20.4)		95 (15.5)	147 (25.4)	
Secondary school	308 (25.9)	332 (27.9)		146 (23.9)	186 (32.2)	
High School	291 (24.5)	359 (30.2)		214 (35.0)	145 (25.1)	
College or Above	214 (18.0)	256 (21.5)		156 (25.5)	100 (17.3)	
Occupation (n, %)			0.03			<0.01
Administrator/other white collar worker	166 (13.9)	155 (13.0)		29 (4.8)	126 (21.8)	
Blue collar worker	226 (19.0)	278 (23.4)		94 (15.4)	184 (31.8)	
Farmer/other	797 (67.0)	755 (63.6)		487 (79.8)	268 (46.4)	
Income (Yuan/month) (n, %)			<0.01			<0.01
<2,000	160 (13.5)	172 (14.5)		69 (11.3)	103 (17.8)	
2,001–5,000	396 (33.3)	441 (37.3)		282 (46.2)	159 (27.5)	
5,001–8,000	333 (28.0)	381 (32.2)		203 (33.2)	178 (30.8)	
>8,001	300 (25.2)	189 (16.0)		57 (9.3)	138 (23.9)	
BMI (kg/m^2^) (mean ± SD)	23.0 ± 3.3	23.0 ± 3.0	0.95	23.5 ± 2.9	22.5 ± 2.9	<0.01
BMI (kg/m^2^) (n, %)			0.22			<0.01
<18.5	93 (7.8)	72 (6.1)		29 (4.8)	43 (7.5)	
18.5–23.9	662 (55.9)	663 (56.1)		318 (52.3)	345 (60.1)	
>24	430 (36.3)	447 (37.8)		261 (42.9)	186 (32.4)	
Regular smoker (n, %)	302 (25.4)	361 (30.4)	<0.01	167 (27.3)	194 (33.6)	0.02
Passive smoking (n, %)	774 (65.1)	513 (43.2)	<0.01	131 (21.5)	382 (66.1)	<0.01
Regular drinker (n, %)	181 (15.2)	162 (13.6)	0.27	67 (11.0)	95 (16.4)	<0.01
Family history of cancer (n, %)	168 (14.1)	88 (7.4)	<0.01	52 (8.5)	36 (6.2)	0.13
Physical activity (n, %)			0.01			<0.01
Less active	621 (52.2)	619 (52.1)		240 (39.3)	379 (65.6)	
Moderate active	383 (32.2)	335 (28.2)		227 (37.2)	108 (18.7)	
More active	185 (15.6)	235 (19.8)		144 (23.6)	91 (15.7)	
Age at menarche, yr, (mean ± SD)^a^	14.5 ± 2.6	14.6 ± 1.9	0.59	14.5 ± 2.0	14.7 ± 1.8	0.17
Menopausal status (n, %)^a^			0.74			<0.01
Premenopausal	153 (29.1)	158 (30.0)		10 (3.8)	148 (56.9)	
Postmenopausal	373 (70.9)	368 (70.0)		256 (96.2)	112 (13.1)	

Continuous variables were evaluated using t-tests or Wilcoxon rank-sum tests. Categorical variables were evaluated using Chi square tests.

^a^Among female subgroup.

**Table 2:** Intakes of energy, fish among case and control subjects in Guangdong, China^a^.

	Cases (n = 1189)			Controls (n = 1189)			*P*_value_
	Mean	SD	Median (25th, 75th)	Mean	SD	Median (25th, 75th)	
Energy (kcal/day)	1591	505.7	1522 (1224, 1863)	1750	537.1	1683 (1367, 2033)	<0.01
Freshwater fish (g/day)	34.54	40.83	23.14 (7.58, 46.85)	46.31	53.84	31.02 (13.54, 58.30)	<0.01
Sea fish (g/day)	23.48	49.95	3.04 (0, 23.96)	21.25	43.24	4.80 (0, 23.03)	0.04
Dried/salted fish (g/day)	3.15	13.74	0 (0, 1.46)	1.59	4.85	0.09 (0, 1.36)	0.92
Shellfish (g/day)	11.71	22.89	4.47 (0.44, 12.74)	10.72	27.45	3.86 (1.05, 11.10)	0.96
Fresh fish (g/day)	65.68	63.35	48.75 (22.70, 85.92)	77.51	74.58	55.60 (32.53, 99.88)	<0.01

Wilcoxon rank-sum test comparing the median consumption levels between cases and controls.

^a^adjusted the fish consumption for total energy intake by the regression residual method.

**Table 3:** Odds ratios (ORs) and 95% confidence intervals (95%CIs) of colorectal cancer according to quartiles of fish intakes.

	Q1	Q2	Q3	Q4	*P*_trend_
Freshwater fish					
No. Cases/Controls	416/296	296/298	264/298	213/297	
Crude OR (95%CI)	1	0.71 (0.57–0.88)	0.63 (0.50–0.79)	0.51 (0.41–0.64)	<0.01
Adjusted OR (95%CI)^a^	1	0.72 (0.57–0.91)	0.59 (0.46–0.75)	0.47 (0.36–0.60)	<0.01
Sea fish					
No. Cases/Controls	376/297	294/298	214/297	305/297	
Crude OR (95%CI)	1	0.78 (0.62–0.97)	0.57 (0.45–0.72)	0.81 (0.65–1.01)	<0.01
Adjusted OR (95%CI)^a^	1	0.77 (0.61–0.98)	0.57 (0.44–0.73)	0.79 (0.62–0.99)	<0.01
Dried/salted fish					
No. Cases/Controls	272/297	395/297	209/298	313/297	
Crude OR (95%CI)	1	1.45 (1.16–1.82)	0.77 (0.60–0.98)	1.15 (0.92–1.45)	0.56
Adjusted OR (95%CI)^a^	1	1.29 (1.01–1.64)	0.80 (0.61–1.03)	1.15 (0.90–1.48)	0.94
Shellfish					
No. Cases/Controls	355/297	210/298	303/297	321/297	
Crude OR (95%CI)	1	0.59 (0.47–0.75)	0.85 (0.68–1.07)	0.90 (0.73–1.13)	0.94
Adjusted OR (95%CI)^a^	1	0.65 (0.51–0.84)	0.89 (0.70–1.13)	0.95 (0.75–1.21)	0.84
Fresh fish					
No. Cases/Controls	418/296	257/298	284/299	230/296	
Crude OR (95%CI)	1	0.61 (0.49–0.76)	0.67 (0.54–0.84)	0.55 (0.44–0.69)	<0.01
Adjusted OR (95%CI)^a^	1	0.54 (0.43–0.69)	0.62 (0.49–0.79)	0.49 (0.38–0.62)	<0.01

^a^Odds ratio was adjusted for age, sex, marital status, education, income level, occupation, family history of cancer, smoking status, passive smoking, alcohol drinking, physical activity and BMI.

**Table 4:** Odds ratios (ORs) and 95% confidence intervals (95%CIs) of colorectal cancer according to quartiles of fish intakes by sex.

	Males (n = 663)					Females^b^ (n = 526)					*P*_interaction_
	Q1	Q2	Q3	Q4	*P*_trend_	Q1	Q2	Q3	Q4	*P*_trend_	
Freshwater fish											
No. Cases/Controls	227/165	171/166	156/166	109/166		189/131	125/132	108/132	104/131		
Crude OR (95%CI)	1	0.75 (0.56–1.00)	0.68 (0.51–0.92)	0.48 (0.35–0.65)	<0.01	1	0.66 (0.47–0.91)	0.57 (0.40–0.80)	0.55 (0.39–0.77)	<0.01	
Adjusted OR (95%CI)^a^	1	0.72 (0.52–0.99)	0.59 (0.43–0.82)	0.41 (0.29–0.58)	<0.01	1	0.68 (0.48–0.98)	0.54 (0.38–0.78)	0.50 (0.34–0.72)	<0.01	0.95
Sea fish											
No. Cases/Controls	174/166	183/166	112/165	194/166		202/131	111/132	102/132	111/131		
Crude OR (95%CI)	1	1.05 (0.78–1.42)	0.65 (0.47–0.89)	1.12 (0.83–1.50)	0.90	1	0.55 (0.39–0.76)	0.50 (0.36–0.70)	0.55 (0.39–0.77)	<0.01	
Adjusted OR (95%CI)^a^	1	0.91 (0.65–1.27)	0.57 (0.40–0.82)	1.02 (0.73–1.41)	0.63	1	0.60 (0.42–0.86)	0.54 (0.38–0.78)	0.59 (0.41–0.84)	<0.01	<0.01
Dried/salted fish											
No. Cases/Controls	164/166	225/165	106/166	168/166		108/131	170/132	103/132	145/131		
Crude OR (95%CI)	1	1.38 (1.03–1.85)	0.65 (0.47–0.90)	1.02 (0.76–1.39)	0.19	1	1.56 (1.11–2.20)	0.95 (0.66–1.36)	1.34 (0.95–1.90)	0.54	
Adjusted OR (95%CI)^a^	1	1.11 (0.81–1.54)	0.65 (0.45–0.92)	1.03 (0.74–1.44)	0.43	1	1.64 (1.13–2.38)	1.11 (0.74–1.65)	1.47 (1.01–2.14)	0.24	0.19
Shellfish											
No. Cases/Controls	176/166	117/166	182/165	188/166		179/131	93/132	121/132	133/131		
Crude OR (95%CI)	1	0.67 (0.48–0.91)	1.04 (0.77–1.40)	1.07 (0.79–1.44)	0.22	1	0.52 (0.36–0.73)	0.67 (0.48–0.94)	0.74 (0.53–1.03)	0.14	
Adjusted OR (95%CI)^a^	1	0.76 (0.54–1.08)	1.11 (0.80–1.54)	1.13 (0.81–1.57)	0.20	1	0.57 (0.39–0.84)	0.69 (0.48–0.99)	0.82 (0.57–1.19)	0.37	0.02
Fresh fish											
No. Cases/Controls	213/165	137/166	177/167	136/165		205/131	120/132	107/132	94/131		
Crude OR (95%CI)	1	0.64 (0.47–0.87)	0.82 (0.61–1.10)	0.64 (0.47–0.87)	0.02	1	0.58 (0.42–0.81)	0.52 (0.37–0.73)	0.46 (0.33–0.65)	<0.01	
Adjusted OR (95%CI)^a^	1	0.54 (0.39–0.75)	0.71 (0.51–0.98)	0.52 (0.37–0.74)	<0.01	1	0.56 (0.39–0.80)	0.51 (0.36–0.74)	0.43 (0.30–0.63)	<0.01	0.04

^a^Odds ratio was adjusted for age, marital status, education, income level, occupation, family history of cancer, smoking status, passive smoking, alcohol drinking, physical activity and BMI.

^b^Odds ratio was adjusted for the various above confounders and menopausal status in female subjects.

**Table 5:** Odds ratios (ORs) and 95% confidence intervals (95%CIs) of colorectal cancer according to quartiles of fish intakes by cancer site.

	Colon cancer (n = 721)					Rectal cancer (n = 468)				
	Q1	Q2	Q3	Q4	*P*_trend_	Q1	Q2	Q3	Q4	*P*_trend_
Freshwater fish										
No. Cases/Controls	228/296	178/298	174/298	141/297		188/296	118/298	90/298	72/297	
Crude OR (95%CI)	1	0.78 (0.60–1.00)	0.76 (0.59–0.98)	0.62 (0.47–0.80)	0.01	1	0.62 (0.47–0.83)	0.48 (0.35–0.64)	0.38 (0.28–0.52)	<0.01
Adjusted OR (95%CI)^a^	1	0.79 (0.60–1.04)	0.70 (0.53–0.92)	0.55 (0.42–0.74)	<0.01	1	0.66 (0.49–0.88)	0.46 (0.33–0.63)	0.40 (0.28–0.55)	<0.01
Sea fish										
No. Cases/Controls	219/297	166/298	129/297	207/297		157/297	128/298	85/297	98/297	
Crude OR (95%CI)	1	0.76 (0.58–0.98)	0.59 (0.45–0.77)	0.95 (0.74–1.21)	0.35	1	0.81 (0.61–1.08)	0.54 (0.40–0.74)	0.62 (0.46–0.84)	<0.01
Adjusted OR (95%CI)^a^	1	0.73 (0.56–0.97)	0.56 (0.42–0.75)	0.91 (0.70–1.20)	0.30	1	0.87 (0.65–1.18)	0.58 (0.42–0.81)	0.66 (0.48–0.91)	0.01
Dried/salted fish										
No. Cases/Controls	165/297	244/297	123/298	189/297		107/297	151/297	86/298	124/297	
Crude OR (95%CI)	1	1.48 (1.15–1.91)	0.74 (0.56–0.99)	1.15 (0.88–1.49)	0.51	1	1.41 (1.05–1.90)	0.80 (0.58–1.11)	1.16 (0.86–1.57)	0.80
Adjusted OR (95%CI)^a^	1	1.32 (1.00–1.74)	0.79 (0.59–1.08)	1.21 (0.91–1.61)	0.85	1	1.25 (0.91–1.70)	0.82 (0.58–1.15)	1.15 (0.84–1.59)	0.98
Shellfish										
No. Cases/Controls	196/297	120/298	199/297	206/297		159/297	90/298	104/297	115/297	
Crude OR (95%CI)	1	0.61 (0.46–0.81)	1.02 (0.79–1.31)	1.05 (0.82–1.35)	0.16	1	0.56 (0.42–0.77)	0.65 (0.49–0.88)	0.72 (0.54–0.97)	0.04
Adjusted OR (95%CI)^a^	1	0.68 (0.50–0.91)	1.02 (0.78–1.35)	1.10 (0.83–1.45)	0.17	1	0.63 (0.46–0.87)	0.73 (0.53–1.00)	0.85 (0.62–1.16)	0.37
Fresh fish										
No. Cases/Controls	211/296	161/298	197/299	152/296		207/296	96/298	87/299	78/296	
Crude OR (95%CI)	1	0.76 (0.58–0.98)	0.92 (0.72–1.19)	0.72 (0.55–0.94)	0.06	1	0.46 (0.34–0.62)	0.42 (0.31–0.56)	0.38 (0.28–0.51)	<0.01
Adjusted OR (95%CI)^a^	1	0.66 (0.50–0.88)	0.81 (0.62–1.07)	0.63 (0.47–0.83)	<0.01	1	0.43 (0.32–0.59)	0.43 (0.32–0.60)	0.38 (0.28–0.53)	<0.01

^a^Odds ratio was adjusted for age, sex, marital status, education, income level, occupation, family history of cancer, smoking status, passive smoking, alcohol drinking, physical activity, BMI.
